# Emergence of the fourth mobile sulfonamide resistance gene *sul4* in clinical *Salmonella enterica*

**DOI:** 10.3389/fmicb.2023.1242369

**Published:** 2023-09-07

**Authors:** Kai Peng, Jianping Deng, Nianli Zou, Xinran Sun, Weifeng Huang, Ruichao Li, Xiaorong Yang

**Affiliations:** ^1^Jiangsu Co-Innovation Center for Prevention and Control of Important Animal Infectious Diseases and Zoonoses, College of Veterinary Medicine, Yangzhou University, Yangzhou, Jiangsu, China; ^2^Institute of Comparative Medicine, Yangzhou University, Yangzhou, Jiangsu, China; ^3^Zigong Center for Disease Control and Prevention, Zigong, Sichuan, China; ^4^Center for Disease Control and Prevention of Sichuan Province, Chengdu, Sichuan, China

**Keywords:** sulfonamide resistance, *sul4*, chromosomally integrated plasmid, *Salmonella enterica*, clinical

## Abstract

The fourth mobile sulfonamide resistance gene *sul4* has been discovered in many metagenomic datasets. However, there is no reports of it in cultured bacteria. In this study, a *sul4* positive clinical *Salmonella enterica* SC2020597 was obtained by conventional *Salmonella* isolation methods and characterized by species identification and antimicrobial susceptibility testing. Meanwhile, the genomic DNA was sequenced using both long-read and short-read methods. Following that, the complete genome was analyzed by bioinformatic methods. The *sul4* gene in *S. enterica* SC2020597 differed from the *sul4* identified in metagenomic data by one amino acid and could confer full resistance to sulfamethoxazole. Genetic location analysis showed that the *sul4* in SC2020597 was carried by a complex chromosomally integrated hybrid plasmid. IS*CR20*-like was strongly associated with the mobilization of *sul4* by core genetic context analysis. To the best of our knowledge, this is the first report of the emergence of *sul4* in clinically cultured *S. enterica*. More important, the *sul4* has the potential to spread to other bacteria with the help of mobile elements.

## Introduction

Sulfonamides are bacteriostatic antimicrobials that inhibit bacterial cellular activity without directly killing the bacteria. They work by interfering with the synthesis of folic acid in bacteria, which is required for the formation of nucleic acids (Skold, 2000; Ovung and Bhattacharyya, 2021). In 1930’s, sulfonamides were firstly introduced for the treatment of human bacterial infections (Skold, 2000; Fernandez-Villa et al., 2019; Nunes et al., 2020). In the years that followed, over 150 sulfonamides and its derivatives were applied in human and veterinary medicine as antibacterial drugs (Baran et al., 2011). Given their extensive utilization, sulfonamide-subsisting bacteria were identified in 2008 for the first time (Dantas et al., 2008), and then many species of bacteria showing sulfonamides resistant were discovered (Deng et al., 2018; Ma et al., 2022). Currently, the most common mechanism of sulfonamide resistance in the majority of bacteria was plasmid-borne, highly mobilized *sul1*, *sul2*, and *sul3*, which encode dihydropteroate synthase (Wang et al., 2014; Nunes et al., 2020; Tang et al., 2022a; Venkatesan et al., 2023). In 2017, the fourth mobile sulfonamide resistance gene *sul4* was identified in river sediment with amplicon metagenomic sequencing for the first time (Razavi et al., 2017). To date, many metagenomic analysis have revealed that *sul4* is already present in a wide range of environmental samples from around the world (Razavi et al., 2017; Marathe et al., 2019; Hutinel et al., 2022). However, it has not yet been identified in cultured bacteria.

Salmonellosis caused by *Salmonella enterica* is a common foodborne diseases frequently occurred around the world (Newell et al., 2010; Jajere, 2019; Tang et al., 2022b). It is estimated that salmonellosis could cause 93.8 million foodborne illnesses and 155 thousand people deaths per year (Majowicz et al., 2010; Eng et al., 2015). The World Health Organization (WHO) has listed it as one of the global health concerns. Furthermore, an antibiotic resistance surveillance of foodborne pathogenic bacteria revealed that the prevalence of antibiotic resistance genes (ARGs) in *Salmonella* was serious, second only to *Escherichia coli* (Jajere, 2019). Many clinically critical ARGs, such as *mcr-1* (Li et al., 2022), *bla*_NDM-5_ (Wang et al., 2020) and *tet*(X4) (Wang et al., 2021), have been identified in *S. enterica* in recently years. Therefore, the public health risk posed by antibiotics resistant *S. enterica* has increasingly arisen. In this study, we identified a fourth mobile sulfonamide resistance gene *sul4* in clinical *S. enterica* for the first time, implying that *Salmonella* could be an important carrier of emerging ARGs.

## Materials and methods

### Bacterial isolate and antimicrobial susceptibility testing

According to previous method (Xia et al., 2009), the *S. enterica* SC2020597 was isolated from a hospital in Guangyuan, Sichuan province, China in 2020. Then, pure cultured SC2020597 was identified as *S. enterica* using matrix-assisted laser desorption ionization time-of-flight mass spectrometry (MALDI-TOF-MS) (Bruker, Bremen, Germany). Meanwhile, the species of isolated *Salmonella* was further confirmed by an online rMLST analysis (Jolley et al., 2018). Subsequently, the minimum inhibitory concentrations (MICs) of SC2020597 was tested by broth microdilution according to Clinical and Laboratory Standards Institute (CLSI) guidelines (https://clsi.org/). *E. coli* ATCC25922 was used for the quality control.

### Conjugation assay and electroporation experiment

In order to investigate the transfer ability of *sul4*, both conjugation assays and electroporation experiments were performed. For the conjugation assay, we used SC2020597 as donor strains and *E. coli* C600 as recipients. The donor and recipient strains were cultured into the logarithmic growth phase with an OD600 value of 0.4 in LB broth, then mixed at a ratio of 1:1 and cultured overnight on LB agar plates. The transconjugants were screened on LB agar plates containing rifampin (300 mg/L) and trimethoprim/sulfamethoxazole (4/76 mg/L). For the electroporation experiment, the genomic DNA of SC2020597 was used as donor DNA, and electrocompetent cells of *S. enterica * ATCC13076 were used as recipients. Electroporation conditions were 200 Ω, 1.8 kV and 25 uF. The transconjugants were screened on LB agar plates containing trimethoprim/sulfamethoxazole (4/76 mg/L). Then, all transconjugants were confirmed by PCR methods targeted at *sul4* and 16S rDNA genes.

### Genomic DNA extraction, sequencing, and cyclic plasmid detection

The genomic DNA of SC2020597 was extracted using FastPure Bacteria DNA Isolation Mini Kit (Vazyme™, China) following the protocol descripting in the manufacturer. The purity and quality of extracted genomic DNA were evaluated using NanoDrop (Thermo ScientificTM) and using a dsDNA High Sensitivity (HS) Assay kit on the Qubit 4 Fluorometer, respectively. Then, 200 μg genomic DNA was sent to GENEWIZ (Suzhou China) to subject short-read sequencing with PE150 strategy on Illumina Hiseq 2,500 platform. Meanwhile, long-read genomic sequencing of SC2020597 was conducted at Oxford Nanopore Technologies MinION platform in our laboratory. Briefly, the long-read sequencing library was prepared using the SQK-RBK109 1D Rapid Barcoding genomic DNA kit according to the user handbook. Then, the prepared library was sequenced with R9.4 flow cells on MinION and the sequencing process was managed with MinKNOW.

The cyclic plasmid form of the chromosomally integrated plasmid was detected using the inverse PCR method with primers cir_F: TTCAGACGGACTGGACATCG and cir_R: GCGGAATTGTTCAGGGGGTA. The genomic DNA of SC2020597 was used as template DNA. The master mix for application in long fragments was used for PCR amplification reaction system.

### Data analysis

The short-read raw reads were filtered to remove low-quality base and adapters using fastp with default parameters (Chen et al., 2018). Then, the complete genome of SC2020597 was generated with a hybrid assembly strategy combining of clean short-read data and long-read data using Unicycler (Wick et al., 2017). Functional annotation of the complete genome was performed by a web-based RAST annotation engine (Aziz et al., 2008; Overbeek et al., 2014; Brettin et al., 2015). Antibiotic resistance genes (ARGs), plasmid replicon genes and insertion sequences (ISs) were identified using abricate tool (https://github.com/tseemann/abricate) based on AMRFinderPlus (Feldgarden et al., 2019), PlasmidFinder (Carattoli et al., 2014) and ISFinder (Siguier et al., 2006) databases, respectively. Multilocus sequence typing (MLST) of the complete bacterial genome was performed using mlst tool (https://github.com/tseemann/mlst). Using *Salmonella* genomes as input data, the web-based application SISTR was used to identify the *Salmonella* serovar (Yoshida et al., 2016). Plasmid comparisons and genetic context comparisons visualization were performed with BRIG (Alikhan et al., 2011) and Easyfig (Sullivan et al., 2011) tools.

### Functional confirmation of *sul4*

To confirm the resistance function of the mutated *sul4*, TA-cloning was performed using a 5 min TA/Blunt-Zero Cloning Kit developed by Vazyme (Vazyme, China). Briefly, the *sul4* gene and its predicted promoter were amplified by PCR using primers sul4_F: TGCCTGCAGGTCGACTCTAGAACCCAAAAGTCTGTAGCCCAAA, sul4_R: ACGGCCAGTGAATTGAGCTCTGGTCTAGTICAAAATCGATCATGT, and then cloned into pUC19 vector. Meanwhile, in order to verify the effect of the base mutation on the function of *sul4*, an unmutated *sul4* recombinant expression plasmid was constructed. Subsequently, the recombinant plasmids were introduced chemically into *E. coli* DH5α. At last, we tested the resistance phenotype of the transconjugants using broth microdilution.

### Data availability

The genome sequences of SC2020597 were deposited into the National Center for Biotechnology information (NCBI) under BioProject PRJNA946266.

## Results and discussion

### Characteristic of the *Salmonella enterica* isolate SC2020597

The isolate SC2020597 was recovered from a clinical patient. We identified it as *S. enterica* using MALDI-TOF-MS and confirmed by Ribosomal Multilocus Sequence Typing (rMLST) analysis. MLST analysis showed that isolate SC2020597 belonged to ST26 *S. enterica*. Serovar analysis classified the isolate as *S. enterica* subsp. *enterica* serovar Thompson, which is one of the most frequent *Salmonella* serovars involved in human infection (Eun et al., 2019). According to previous investigations, the prevalence of *S.* Thompson in clinical patients and food in China was 3.9 and 5.4%, respectively (Wang et al., 2017; Fan et al., 2020). The serovar has the potential to cause outbreaks of *Salmonella* infection. Antimicrobial susceptibility testing showed that the isolate was resistant to multiple antibiotics including kanamycin, ampicillin, tetracycline, sulfamethoxazole and trimethoprim/sulfomethoxazole, but sensitive to aztreonam, meropenem, ciprofloxacin, colistin and enrofloxacin (Supplementary Table S1).

### Functional analysis of *sul4*

The draft genome of SC2020597 was obtained by short-read genome assembly. Many ARGs, plasmid replicon genes and ISs were identified in the draft genome. Of note, we found a *sul4* gene in isolate SC2020597, which had previously only been found in metagenomes (Razavi et al., 2017; Marathe et al., 2019; Hutinel et al., 2022). The *sul4* gene in SC2020597 showed 100% coverage and 99.88% nucleic acid identity to *sul4* (NG_056174). The one nucleic acid substitution of *sul4* causes one amino acid change (W120R). To verify the function of the novel *sul4*, the intact *sul4* gene and its promoter were cloned into pUC19 vector and introduced into *E. coli* DH5α. The *sul4* positive transconjugants were resistant to trimethoprim/sulfomethoxazole and had a MIC for trimethoprim/sulfomethoxazole that was more than 16-fold higher than *E. coli* DH5 with an empty vector (≤ 1/19 mg/L to >32/608 mg/L). Meanwhile, unmutated *sul4*-bearing *E. coli* DH5 also showed full resistance to trimethoprim/sulfomethoxazole (>32/608 mg/L). This demonstrated that W120R substitution had no effect to the function of *sul4*.

### Genomic feature of SC2020597

To decipher the genomic structure feature of SC2020597, long-read sequencing was performed. Then, the complete genome of SC2020597 was generated by a hybrid assembly strategy using short-read and long-read data. The isolate harbored one chromosome with a length of 5, 035, 375 bp and two plasmids, pSC2020597_48k with a length of 48, 530 bp, and pSC2020597_5k with a length of 5, 754 bp. Plasmid replicon analysis showed that pSC2020597_48k was an IncFII type plasmid and pSC2020597_5k was a Col type small plasmid. Of note, no ARG was found in the two plasmids. Online blastn analysis showed that many plasmids from *Salmonella* were similar to pSC2020597_48k, indicating that such plasmids were common in *Salmonella*. Plasmid pSC2020597_5k belonged to a group of small plasmids with a broad host range that were widely distributed in Enterobacteriaceae.

**Figure 1:**
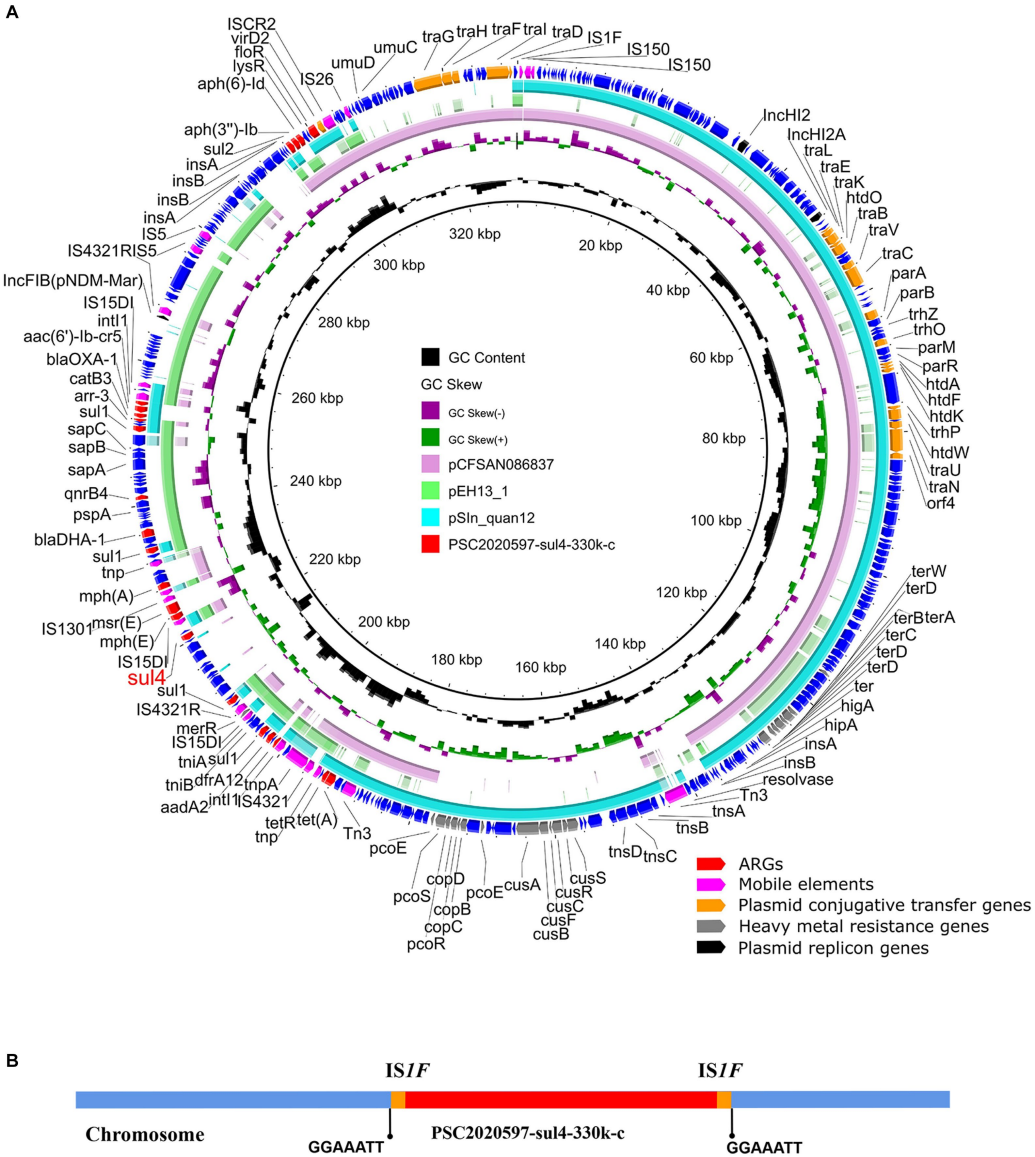
Structure analysis of *sul4*-bearing chromosomally integrated plasmid in *Salmonella enterica*. **(A)** Comparative analysis of chromosomally integrated plasmid PSC2020597-sul4-330 k-c in this study with other similar plasmids including pSIn_quan12 (ON960352.1), pEH13_1 (CP089098.1), and pCFSAN086837 (CP039438.1). **(B)** The integration sites of chromosomal plasmids PSC2020597-sul4-330 k-c.

A total of 22 ARGs, including *sul4*, were detected in SC2020597, and all of them were located on chromosome (Supplementary Table S2). In addition, three plasmid replicon genes were discovered on the chromosome of SC2020597. Further analysis found that a multi-drugs resistant plasmid was integrated into chromosome of SC2020597. The chromosomally integrated plasmid was 330, 232 bp in length and was designated as PSC2020597-sul4-330 k-c (Figure 1A). The phenomenon that plasmid was integrated into chromosome have been frequently reported (Muthuirulandi Sethuvel et al., 2021; Shigemura et al., 2021; Chang et al., 2022), especially in *Salmonella*, and is commonly mediated by homologous recombination of ISs. We found that PSC2020597-sul4-330 k-c was flanked by IS*1F* on chromosome of SC2020597. Meanwhile, a 7 bp direct repeat sequence was found around PSC2020597-sul4-330 k-c (Figure 1B). These findings provided strong evidences that PSC2020597-sul4-330 k-c was integrated into chromosome via the homologous recombination of IS*1R*. The precursor of PSC2020597-sul4-330 k-c was a hybrid plasmid with complex structure. Half of PSC2020597-sul4-330 k-c was composed by a typical IncHI2/HI2A plasmid. Another half was made up of flexible genetic arrays. Blastn analysis with NCBI nr database showed that PSC2020597-sul4-330 k-c was most closely related to pSIn_quan12 (GenBank: ON960352.1), which had 66% coverage and 99.98% identity to PSC2020597-sul4-330 k-c (Figure 1A). Meanwhile, many other plasmids similar to PSC2020597-sul4-330 k-c were also found in nr database, and the complete structure of PSC2020597-sul4-330 k-c could almost be covered by the genetic array of these plasmids (Figure 1A). Hence, PSC2020597-sul4-330 k-c was most likely formed through the recombination of diversity of plasmids and then integrated into chromosome. The discovery of *sul4* in a complex chromosomally integrated plasmid implied that *sul4* gene had already appeared and spread in plasmids. We should take notice to monitor the spread of *sul4* by plasmids or other mobile genetic elements.

### The transfer ability of *sul4*

By the genetic analysis, we found that *sul4* was located on a chromosomally integrated plasmid. However, a previous study demonstrated that chromosomally integrated plasmids could also be transferred to other recipients by conjugation assay (Chang et al., 2022). Here, we verified that there is a circle form of chromosomally integrated plasmid PSC2020597-sul4-330 k-c in some clones of SC2020597 using the inverse PCR method and confirmed by Sanger sequencing (Supplementary Figure S1). This phenomenon implied that the chromosomally integrated plasmid PSC2020597-sul4-330 k-c had potential to horizontal transfer by conjugation. Subsequently, we used *E. coli* C600 as recipients to test the transfer of *sul4* by conjugation assay. The *sul4* gene could not be transferred into *E. coli* C600 after multiple tries. Then, we attempted to verify the transfer ability of *sul4* using electroporation experiments with *S. enterica * ATCC13076 as recipients. The *sul4*-positive *S. enterica* ATCC13076 was successfully screened and showed resistance to trimethoprim/sulfomethoxazole.

### The core genetic structure of *sul4*

**Figure 2:**
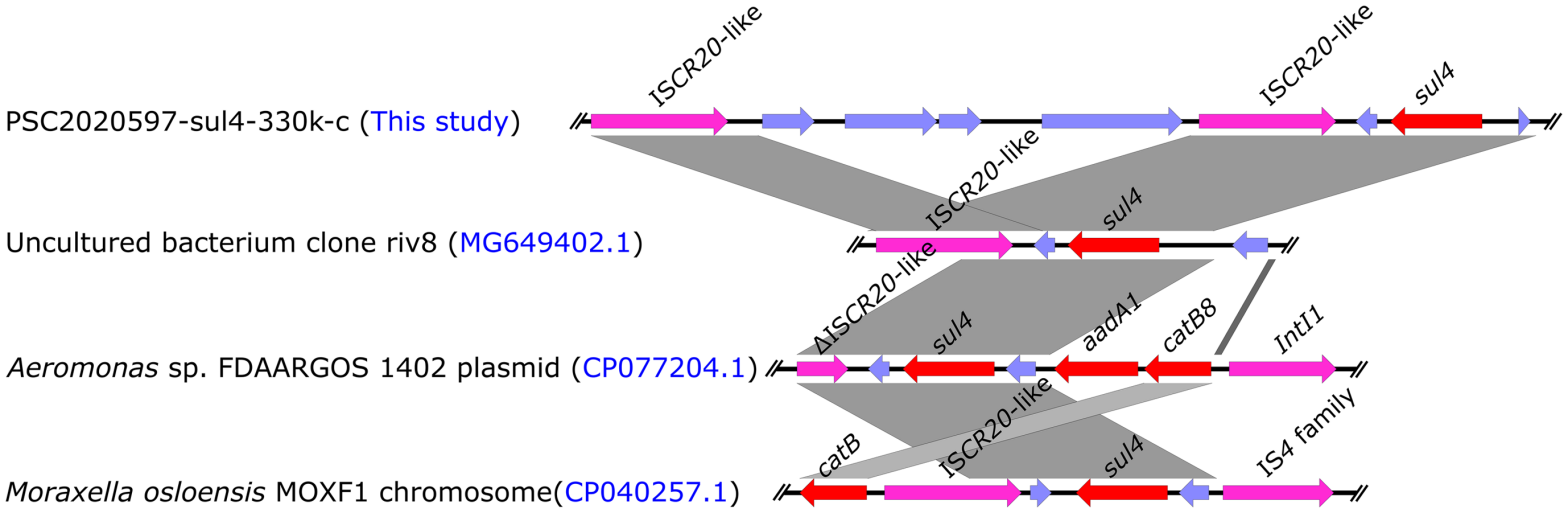
Linear comparison of the core genetic structures of *sul4* gene. Comparative analysis of the genetic context of *sul4* in PSC2020597-sul4-330 k-c with the genetic contexts of *sul4* in uncultured riv8 (MG649402.1), isolates FDAARGOS 1402 (CP077204.1), and MOXF1 (CP040257.1).

Mobile elements played a key role in the dissemination of ARGs (Partridge et al., 2018). Previous research discovered an IS*CR* family transposase IS*CR20*-like (GneBank: MG649402.1) downstream of *sul4* that could be mobilized along with their adjacent genes via rolling-circle transposition without the assistance of any other transposase protein (Razavi et al., 2017). Therefore, *sul4*-IS*CR20*-like was considered to be mobilizable as an entire integrin (Razavi et al., 2017). Subsequently, we investigated the core genetic structure of *sul4* in PSC2020597-sul4-330 k-c. We also found an IS*CR20*-like in the downstream of *sul4*, which was consistent with previous structure of *sul4* in metagenome (GneBank: MG649402.1) (Figure 2). In addition, other *sul4*-bearing genetic contexts associated with IS*CR20*-like or truncated IS*CR20*-like were also found in other bacterial chromosome or plasmid in NCBI nr database (Figure 2). These findings demonstrated that the dissemination of *sul4* was probably driven by IS*CR20*-like. Although the transfer of *sul4*-IS*CR20*-like has not been verified by experiment, other similar transfer events have been confirmed, such as *tet*(X4)-IS*CR2* (He et al., 2019). Hence, the mobilization of *sul4*-IS*CR20*-like was likely to happen and promoted the dissemination of *sul4*. Of note, *sul4* was found to be integrated into a class 1 integron in the plasmid of *Aeromonas* sp. FDAARGOS 1402 (Figure 2). It means that novel mobile genetic structure harboring *sul4* is emerging, which may accelerate the propagation of *sul4*. Therefore, we should take more attention to the surveillance of *sul4* in order to slow its spread.

## Conclusion

To our best knowledge, this is the first report of the emergence of *sul4* in clinically cultured *S. enterica*. The genetic location analysis showed that *sul4* had already emerged in common and continually evolving hybrid plasmids of Enterobacteriaceae. Meanwhile, we found that IS*CR20*-like played an important role in the spread of *sul4*. These findings demonstrated that *sul4* has the potential to be prevalent in various bacteria, and further reduce the effect of sulfonamide.

## Data availability statement

The datasets presented in this study can be found in online repositories. The names of the repository/repositories and accession number(s) can be found at: https://www.ncbi.nlm.nih.gov/, PRJNA946266.

## Author contributions

KP, JD, and NZ: conception and design. KP, JD, NZ, and XS: methodology. WH: collection and assembly of data. KP and WH: data analysis and interpretation. KP: writing—original draft. RL and XY: writing—reviewing and editing. All authors contributed to the article and approved the submitted version.

## Funding

This work was supported by the Sichuan Science and Technology Program (2022ZDZX0017), Postgraduate Research & Practice Innovation Program of Jiangsu Province (SJCX21_1631) and the Priority Academic Program Development of Jiangsu Higher Education Institutions (PAPD).

## Conflict of interest

The authors declare that the research was conducted in the absence of any commercial or financial relationships that could be construed as a potential conflict of interest.

## Publisher’s note

All claims expressed in this article are solely those of the authors and do not necessarily represent those of their affiliated organizations, or those of the publisher, the editors and the reviewers. Any product that may be evaluated in this article, or claim that may be made by its manufacturer, is not guaranteed or endorsed by the publisher.
