# Supplementary material for: Emergence of the fourth mobile sulfonamide resistance gene sul4 in clinical Salmonella enterica
Source: Front Microbiol. 2023 Sep 7;14:1242369. doi: 10.3389/fmicb.2023.1242369 (PMC10512727; doi:10.3389/fmicb.2023.1242369)
Supplement: Supplementary file 1 [file Data_Sheet_1.docx]

**Supplementary data**

Supplementary Table 1. MIC^a^ values (mg/L) of different antimicrobials of SC2020597.

| Isolates | antibiotics^b^ | | | | | | | | | |
| --- | --- | --- | --- | --- | --- | --- | --- | --- | --- | --- |
| / | AMP | TET | KAN | CL | CIP | ENR | SMZ | SXT | MEM | ATM |
| Resistance breakpoints | ≥32 | ≥16 | ≥64 | >2 | ≥1 | / | ≥16 | ≥4/76 | ≥4 | ≥16 |
| SC2020597 | >128 | 16 | >128 | 0.5 | ≤0.25 | ≤0.25 | 32 | >32/608 | ≤0.25 | ≤0.25 |
| ATCC25922 | 4 | ≤0.5 | 0.5 | ≤0.25 | 1 | ≤0.25 | ≤0.5 | ≤0.25/0.475 | ≤0.25 | ≤0.25 |

^a^ MIC minimum inhibitory concentrations

^b^ AMP ampicillin, TET tetracycline, KAN kanamycin, CL colistin, CIP ciprofloxacin, ENR enrofloxacin, SMZ sulfamethoxazole, SXT trimethoprim-sulfomethoxazole, MEM meropenem, ATM, aztreonam.

Supplementary Table 2. ARGs detected in isolate SC2020597.

| Resistance gene | Identity (%) | Coverage (%) | Position in contig |
| --- | --- | --- | --- |
| *aph(6)-Id* | 100 | 100 | 1948697..1949533 |
| *aph(3'')-Ib* | 100 | 100 | 1947894..1948697 |
| *aadA2* | 100 | 100 | 1852918..1853709 |
| *aac(6')-Ib-cr* | 100 | 100 | 1904701..1905219 |
| *aac(6')-Iaa* | 97.72 | 100 | 4244427..4244864 |
| *bla*_DHA-1_ | 100 | 100 | 1886405..1887544 |
| *bla*_OXA-1_ | 100 | 100 | 1903740..1904570 |
| *msr*(E) | 100 | 100 | 1875071..1876546 |
| *mph*(A) | 99.89 | 100 | 1878873..1879778 |
| *mph*(E) | 100 | 99.10 | 1874139..1875015 |
| *floR* | 98.19 | 99.92 | 1950490..1951703 |
| *catB3* | 100 | 100 | 1902970..1903602 |
| *qnrB4* | 100 | 100 | 1891668..1892312 |
| *arr-3* | 100 | 100 | 1902433..1902885 |
| *sul4* | 99.88 | 100 | 1871015..1871877 |
| *sul1* | 100 | 100 | 1854214..1855053 |
| *sul1* | 100 | 100 | 1901030..1901869 |
| *sul1* | 100 | 100 | 1883989..1884828 |
| *sul1* | 100 | 100 | 1860134..1860973 |
| *sul2* | 100 | 100 | 1947018..1947833 |
| *tet*(A) | 100 | 100 | 1842390..1843589 |
| *dfrA12* | 100 | 100 | 1852013..1852510 |


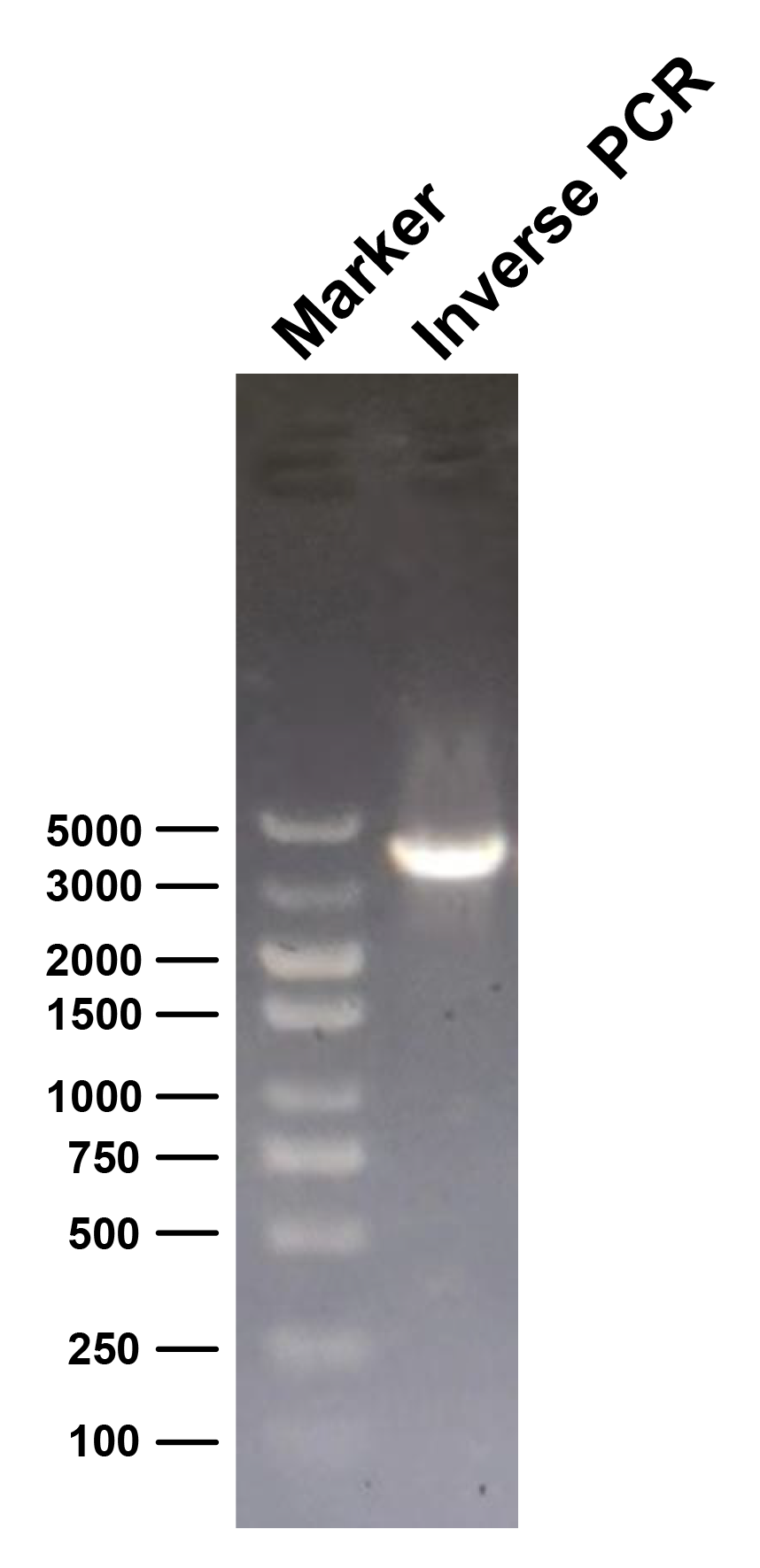


Supplementary Figure 1. Gel electrophoresis of inverse PCR products generated by primers cir_F and cir_R.
